# Head-to-head comparison of two human papillomavirus vaccines for efficacy against cervical intraepithelial neoplasia grade 3 and adenocarcinoma *in situ*—population-based follow-up of two cluster-randomized trials

**DOI:** 10.3389/fcimb.2024.1437704

**Published:** 2024-09-09

**Authors:** Matti Lehtinen, Penelope Gray, Tapio Luostarinen, Tiina Eriksson, Dan Apter, Anne Bly, Katja Harjula, Kaisa Heikkilä, Mari Hokkanen, Marjo Kuortti, Pekka Nieminen, Mervi Nummela, Jorma Paavonen, Johanna Palmroth, Tiina Petäjä, Ville N. Pimenoff, Eero Pukkala, Joakim Dillner

**Affiliations:** ^1^ Department of CLINTEC, Karolinska Institute, Stockholm, Sweden; ^2^ Faculty of Medicine, Tampere University, Tampere, Finland; ^3^ Institute for Epidemiological Cancer Research, Finnish Cancer Registry, Helsinki, Finland; ^4^ Youth Clinic, VL-Medi, Helsinki, Finland; ^5^ Department of Obstetrics and Gynecology, University of Helsinki, Helsinki, Finland; ^6^ Department of Obstetrics and Gynecology, University of East-Finland, Kuopio, Finland

**Keywords:** cervical neoplasia, follow-up, human papillomavirus, vaccine efficacy, randomized trial

## Abstract

**Introduction:**

We report head-to-head comparison of the bivalent and quadrivalent HPV vaccine efficacies against immediate precursors of cervical cancer from 15 years’ country-wide cancer registry follow-up of phase III trial cohorts and an age-aligned cohort of unvaccinated women.

**Methods:**

These individually and/or clusterrandomized cohorts of HPV6/11/16/18- and HPV16/18-vaccinated and unvaccinated women were enrolled, respectively, in 2002, 2004, and 2003/2005. The trial cohorts comprised initially 16- to 17-year-old HPV6/11/16/18-vaccinated FUTURE II (NCT00092534) participants (866) and HPV16/18-vaccinated PATRICIA (NCT00122681) and 012 trial (NCT00169494) participants (2,465), and 16,526 initially 16- to 19-year-old unvaccinated controls. After active 4-year clinical follow-up, passive, country-wide Finnish Cancer Registry (FCR) follow-up for cervical intraepithelial neoplasia grade 3 (CIN3) and adenocarcinoma in situ (AIS) was based on consented use of unique personal identifiers and started 6 months after the end of the FUTURE II and PATRICIA trials in 2007 and 2009, and ended at the end of 2019. The follow-up with altogether 229,020 follow-up years was age-aligned to ensure that similarly aged cohorts were passively followed up for 15 years post=vaccination for the intention-to-treat analyses of vaccine efficacy.

**Results:**

Overall, we identified 5 and 16 CIN3 (no AIS) cases in the HPV6/11/16/18 and HPV16/18 cohorts, respectively, during the FCR-based follow-up. In the unvaccinated cohort, we identified 281 CIN3 cases, 20 AIS cases, and 13 cases with invasive cervical cancer. Vaccine efficacies against CIN3+ were 68.4% and 64.5% for the quadrivalent and the bivalent vaccines, respectively, with overlapping confidence intervals.

**Discussion:**

Long-term follow-up of randomized, initially adolescent HPV-vaccinated and unvaccinated cohorts shows, in this head-to-head setting, that the bivalent and quadrivalent HPV vaccines are equally effective against immediate precursors of cervical cancer.

## Introduction

Head-to-head comparisons of the licensed bi-, quadri-, and nonavalent human papillomavirus (HPV) vaccines for safety and immunogenicity have been mostly restricted to the linkages of vaccinated cohorts with population-based health registers for new-onset potential immune-mediated disorders (pIMDs) (Arnheim et al., 2013; Lehtinen et al., 2016) or follow-up of vaccine-induced serum antibody levels exploiting a consent-based biobank (Artemchuk et al., 2019; Kann et al., 2021; Mariz et al., 2021; Arroyo et al., 2022). While the former has indicated no safety concerns, the latter documented low type-specific HPV antibody levels in the quadri- and nonavalent vaccine recipients as compared to the bivalent vaccine recipients (Artemchuk et al., 2019; Kann et al., 2021; Mariz et al., 2021). Furthermore, 10% of the quadrivalent but not the bivalent vaccine recipients lack both total and neutralizing HPV18 L1 antibodies 12 years post-vaccination (Artemchuk et al., 2019; Mariz et al., 2021).

Whether or not the abovementioned differences in vaccine-induced antibody levels have an impact on vaccine efficacy (VE) is open. Comparison of the phase III trial data for per protocol cohorts of initially HPV-negative women suggested a significant difference in 4-year VE against cervical intraepithelial neoplasia grade 3 (CIN3) between the bivalent (93.2%) and the quadrivalent vaccine (43.0%) (Muñoz et al., 2010; Lehtinen et al., 2012; Lehtinen and Dillner, 2013). However, baseline differences, with the bivalent vaccine recipients having been high-risk (hr) HPV negative and the quadrivalent vaccine recipients having been merely HPV16/18 negative at the baseline, probably explain the observed difference. On the other hand, long-term follow-up of a per-protocol phase III trial cohort in the Scandinavian countries has reported sustainable, close to 100% VE against CIN3 for the quadrivalent vaccine up to 14 years post-vaccination (Kjaer et al., 2020), while follow-up of our intention-to-treat (ITT) III trial cohort reported for the bivalent vaccine sustainable 66% and 100% VEs against CIN3 and invasive HPV cancers, respectively, after 10 and 15 years (Lehtinen et al., 2017a, 2021).

For head-to-head comparison of VEs against CIN3+, we linked cohorts of bi- or quadrivalent vaccine recipients and unvaccinated women with Finnish Cancer Registry (FCR) 15 years post-vaccination.

## Methods

### Recruitment

**Figure 1:**
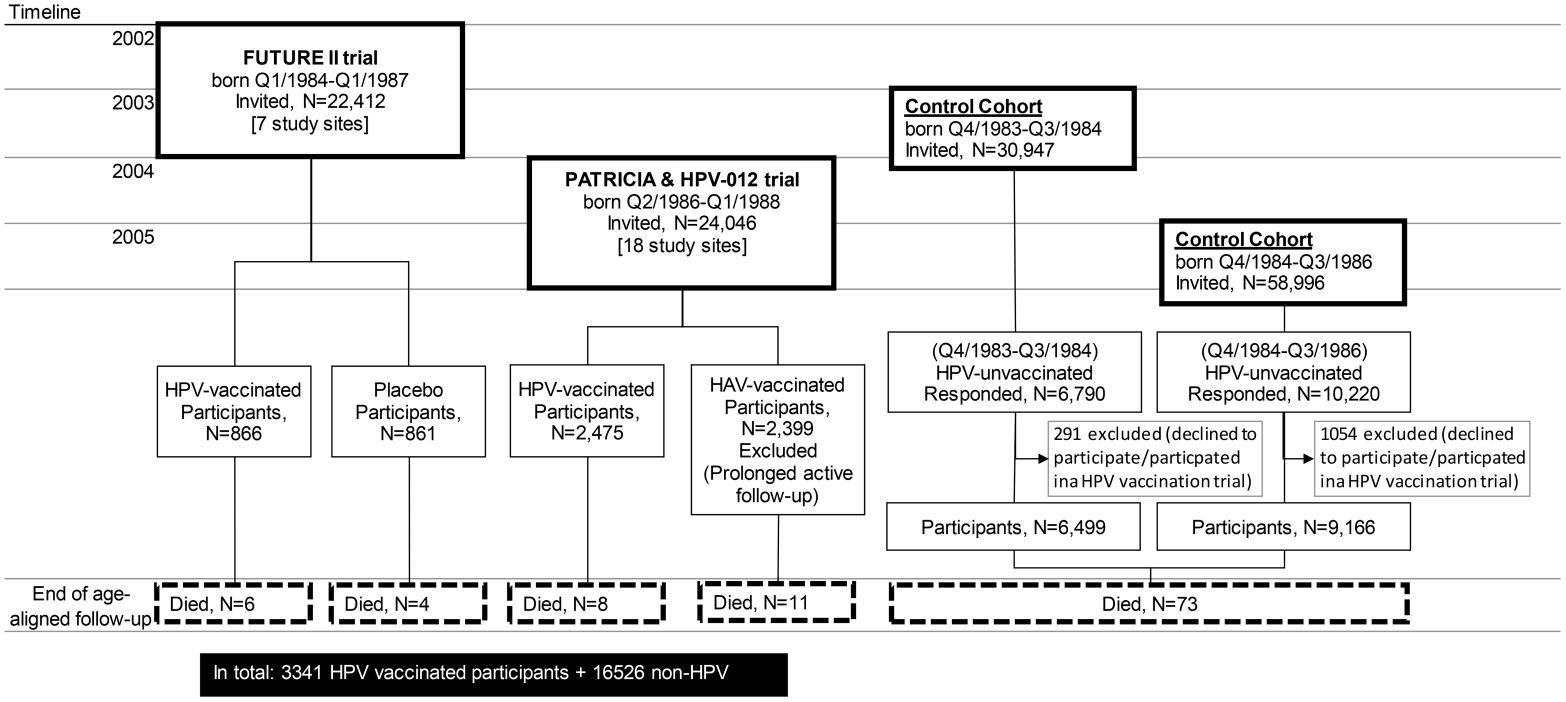
Study flowchart.

All 22,412 female patients born in the fourth quarter of 1984 (Q4/1984) to Q1/1987 were invited to participate in the FUTURE II (NCT00092534) [13] phase III HPV6/11/16/18 vaccine trial at the age of 16 to 17 years in seven cities in Q4/2002–Q1/2003. All 24,064 female patients born Q2/1986–Q1/1988 were invited to participate in the PATRICIA (NCT00122681) phase III trial or the HPV-012 (NCT00337818) (Lehtinen et al., 2006a, 2017a) phase II trial also at the age 16 to 17 years in 18 cities in Q2/2004–Q1/2005 (**Figure 1**). Altogether, there were 3,341 16- to 17-year-old HPV-vaccinated participants by trial: 866, 2,409, and 66 (**Figure 1**).

In June 2003 and June 2005, all (30,947 and 58,996) 18- to 19-year-old Finnish female patients from the entire, immediately adjacent birth cohorts born in Q3/1984–Q2/1985 and Q3/1985–Q2/1987, respectively, not eligible to the abovementioned clinical trials, were invited to participate in FCR-based follow-up (**Figure 1**) (Lehtinen et al., 2006a, 2006b, 2006c). Earlier HPV vaccination was an exclusion criterion. In June 2003 and June 2005, 6,499 and 9,166 18- to 19-year-old non-HPV-vaccinated female patients were enrolled, respectively (**Figure 1**). During Q4/2002–Q1/2003, an additional 861 16- to 17-year-old placebo recipients of the FUTURE II trial were enrolled (**Figure 1**).

All clinical trial participants and non-HPV-vaccinated controls consented to the FCR-based follow-up for invasive, histopathologically confirmed cancer outcome (Lehtinen et al., 2006a, 2006b). It was based on unique Finnish personal identifiers available at our study database following the informed consent and generally used at the FCR. It was age-aligned (Rana et al., 2013) to ensure that all the originally 16- to 17-year-old HPV-vaccinated women and the originally 16- to 19-year-old non-HPV-vaccinated women were of similar age during comparable follow-up time periods between 2007 and 2020, which provided 9,567 (FUTURE II cohort), 27,222 (PATRICIA cohort), and 19,2231 follow-up years.

All study participants of the FCR-based follow-up were invited to cervical screening at the ages of 25, 30, and 35 years. For those with abnormal cytology during the trials, there was referral to colposcopy with biopsy within 6 months and the FCR follow-up thus started 6 months after the trials’ end. All the cohorts were invited to respond at the age of 22 years to a lifestyle questionnaire, with special emphasis on sexual health and previous HPV vaccination.

Death and emigration were elimination criteria. For the non-HPV-vaccinated individuals, HPV vaccination was an elimination criteria. All the clinical HPV vaccination trials and the long-term follow-up of the trial cohorts and the non-HPV-vaccinated cohort (NCT01393470) were approved by the Finnish National Ethical Review Board (ERB, TUKIJA 1150/2002, 1153/2003, and 1174/2004).

Post-vaccination serum samples for all the different trial participants, in total 358 quadrivalent vaccine recipients and 861 bivalent vaccine recipients, were retrieved from the Finnish Maternity Cohort Serum bank between 2003 and the end of 2016 (Lehtinen et al., 2017b; Mariz et al., 2021). For the quadrivalent and bivalent vaccine recipients, follow-up time-stratified (2-4, 5-7, 8-10, 11-12 years) random selection of serum samples resulted in the availability of 339 and 341 matched post-vaccination serial serum samples during up to 12 years of follow-up.

### Laboratory analyses

Diagnostic histopathological blocks were requested from the pathology laboratories that had notified the FCR about the incident cancer cervical neoplasia cases, according to a specific permission from the Finnish National Supervisory Authority for Welfare and Health (Valvira) without informing the patients. The presence of neoplastic tissue was reviewed by two experienced pathologists (Lehtinen et al., 2017b, 2021).

A pseudovirion-based assay was used to determine neutralizing antibodies to HPV16 and 18 as previously described (Mariz et al., 2021). Serum dilutions inhibiting (neutralizing) 50% of the luciferase activity (EC_50_ values) were calculated, and EC_50_ values greater than 40 for HPV16 and HPV18, respectively, corresponding to 1.3 and 1.1 International Units/mL were defined as positive.

### Statistical analyses

Overall, the enrolled HPV-vaccinated and unvaccinated adolescents provided over 80% power for the identification of VE against CIN3+ (Lehtinen et al., 2017b). The VE was calculated according to the ITT principle including all individuals (regardless of baseline HPV status), more than 95% of whom had received three vaccine doses in the HPV-vaccine arm. The statistical software SAS 9.4 (SAS Institute, Cary, NC) was used; 95% confidence intervals (95% CIs) were based on the exact binomial distribution of the number of vaccinated cases conditional on the total number of cases (Ewell, 1996).

We calculated Spearman non-parametric correlation coefficients (*r*s) of HPV16 and HPV18 neutralizing antibody levels by vaccine overall and quartile-wise using R (version 4.0.0).

## Results

In FUTURE II and PATRICIA trials, there were 866 and 2,475 Finnish, initially 16- to 17-year-old participants who received three doses of either the quadrivalent or the bivalent vaccine, respectively, in 2002–2003 and 2004–2005 (**Figure 1**). Concomitant control cohorts of unvaccinated 18- to 19-year-old women were enrolled in 2003 (6,499 participants) and in 2005 (9,166 participants). In addition, the 861 16- to 17-year-old Finnish placebo recipients of the FUTURE II trial were included in the control cohort.

The different cohorts were age-aligned and passively followed for 15 years with the FCR-linkage up to the end of 2020, or until the diagnosis of CIN3+, emigration, or death. The total numbers of passive follow-up years were 9,567, 27,222, and 192,231 for the Finnish FUTURE II, PATRICIA, and concomitant control cohorts, respectively. Altogether, five CIN3+ cases (one in the bivalent vaccine recipient cohort and four in the unvaccinated control cohort) were identified due to the active, semi-annual clinical trial follow-up, or in the control cohort, up to 4.5 years post-vaccination were excluded from the passive FCR-based follow-up (**Table 1**). During the passive follow-up, the incidence rates of CIN3 and CIN3+ were approximately two times and two to four times lower, respectively, in the quadrivalent and in the bivalent vaccine recipients as compared to the unvaccinated control cohort (**Table 1**), albeit with overlapping 95% CIs.

**Table 1:** Number and incidence (rate/10,000 women years) of cervical neoplasia grade 3 (CIN3) or worse (CIN3+) lesions identified at linkage with the Finnish Cancer Registry 4 to 17 years post-human papillomavirus (HPV) vaccination with three doses of the quadrivalent (HPV6/11/16/18) or the bivalent (HPV16/18) vaccine in baseline 16- to 17-year-old women and unvaccinated, age-aligned controls between 2002 and 2020.

HPV-vaccine cohorts					Unvaccinated cohort	
Follow-up time	HPV6/11/16/18	(N=866)	HPV16/18(N=2465)		(N=16526)	
(years)	n (Rate)	[95% CI]	n (Rate)	[95% CI]	n (Rate)	[95% CI]
CIN3)						
4.0 4.4	-(-)	[-]	1 (8.9)	[4.9, 16]	5 (5.9)	[2.5, 14]
4.5 9.4	- (-)	[-]	4 (3.3)	[1.2, 8.7]	86.(9.9)	[8.0, 12]
9.5 14.4	4 (9.3)	[3.5,25]	11 (9.0)	[5.0, 16]	159 (18.3)	[16,21]
14.5 -	1 (10.7)	[1.5, 76]	1 (3.8)	[0.5, 27]	31 (17.3)	[12, 25]
CIN3+)						
4.0-4.4	-(-)	[-]	1 (8.9)	[4.9, 8.9]	5 (5.9)	[2.5.14]
4.5-9.4	-(-)	[-]	4 (3.3)	[1.2, 8,7]	94*(11.1)	[9.1,14]
9.5 14.4	4 (9.3)	[3.5, 25]	11 (9.0)	[5.0, 16]	181 (20.9)	[18,24]
14.5-	1 (10.7)	[1.5, 76]	1 (3.8)	[0.5, 27]	349(19.0)	[14,27]

*3 cases with adenocarcinoma *in situ*, 1 case with adenocarcinoma, and 4 cases with squamous cell carcinoma.

^#^14 cases with adenocarcinoma *in situ*, 2 cases with adenocarcinoma, and 6 cases with squamous cell carcinoma.

^§^3 cases with adenocarcinoma *in situ*.

For the overall passive follow-up time of 15 years, the ITT VE estimates against CIN3 and against CIN3+ were essentially identical for the quadrivalent vaccine (VE_CIN3_ = 64.6% and VE_CIN3+_ = 68.4%) and the bivalent vaccine (VE_CIN3_ = 59.8% and VE_CIN3+_ = 64.5%) with widely overlapping CIs (**Table 2**). We noted some variation in the VEs over the different 5-year periods of the passive follow-up probably due to limited numbers of vaccinees (**Table 2**).

**Table 2:** Vaccine efficacy against cervical neoplasia grade 3 (CIN3) or worse (CIN3+) lesions between 4 and 17 years post-human papillomavirus (HPV) vaccination with three doses of the quadrivalent (HPV6/11/16/18, *N* = 866) or the bivalent (HPV16/18, *N* = 2,465) vaccine in baseline 16- to 17-year-old women compared to unvaccinated, age-aligned controls (*N* = 16,526) between 2002 and 2020.

Follow-up (years)	HPV6/11/16/18			Vaccine Efficacy	[95%CI]	HPV/16/18			Vaccine Efficacy	[95%CI]
	Group	Cases (n)	(Rate)			Group	Cases (n)	(Rate)		
CIN3)										
4.5 - 9.4	Vaccine	–	(-)	100%	[29,100]	Vaccine	4	(3.3)	67.0%	[10,88]
	Control	86	(9.9)			Control	86	(9.9)		
9.5 - 14.4	Vaccine	4	(9.3)	49.1%	[-37,81]	Vaccine	11	(9.0)	50.9%	[9.6, 73]
	Control	159	(18.3)			Control	159	(18.3)		
14.5 -	Vaccine	1	(10.7)	38.1%	[-354,92]	Vaccine	1	(3.8)	78.3%	[-59, 97]
	Control	31	(17.3)			Control	31	(17.3)		
Total	Vaccine	5	(5.2)	64.6%	[21, 99]	Vaccine	16	(5.8)	59.8%	[34, 76]
	Control	276	(14.6)			Control	276	(14.6)		
CIN3+)										
4.5 - 9.4	Vaccine	–	(-)	100%	[37, 100]	Vaccine	4	(3.3)	70.7%	[20,89]
	Control	94*	(11.1)			Control	94*	(11.1)		
9.5 - 14.4	Vaccine	4	(9.3)	55.6%	[-20, 84]	Vaccine	11	(9.0)	57.1%	[21, 77]
	Control	181#	(20.9)			Control	181^#^	(20.9)		
14.5 -	Vaccine	1	(10.7)	43.5%	[-312, 92]	Vaccine	1	(3.8)	80.2%	[-45, 97]
	Control	34	(19.0)			Control	34^§^	(19.0)		
Total	Vaccine	5	(5.2)	68.4%	[24, 87]	Vaccine	16	(5.8)	64.5%	[41, 79]
	Control	309	(16.5)			Control	309	(16.5)		

*3 cases with adenocarcinoma *in situ*, 1 case with adenocarcinoma, and 4 cases with squamous cell carcinoma.

^#^14 cases with adenocarcinoma *in situ*, 2 cases with adenocarcinoma, and 6 cases with squamous cell carcinoma.

^§^Three cases with adenocarcinoma *in situ*.

**Figure 2:**
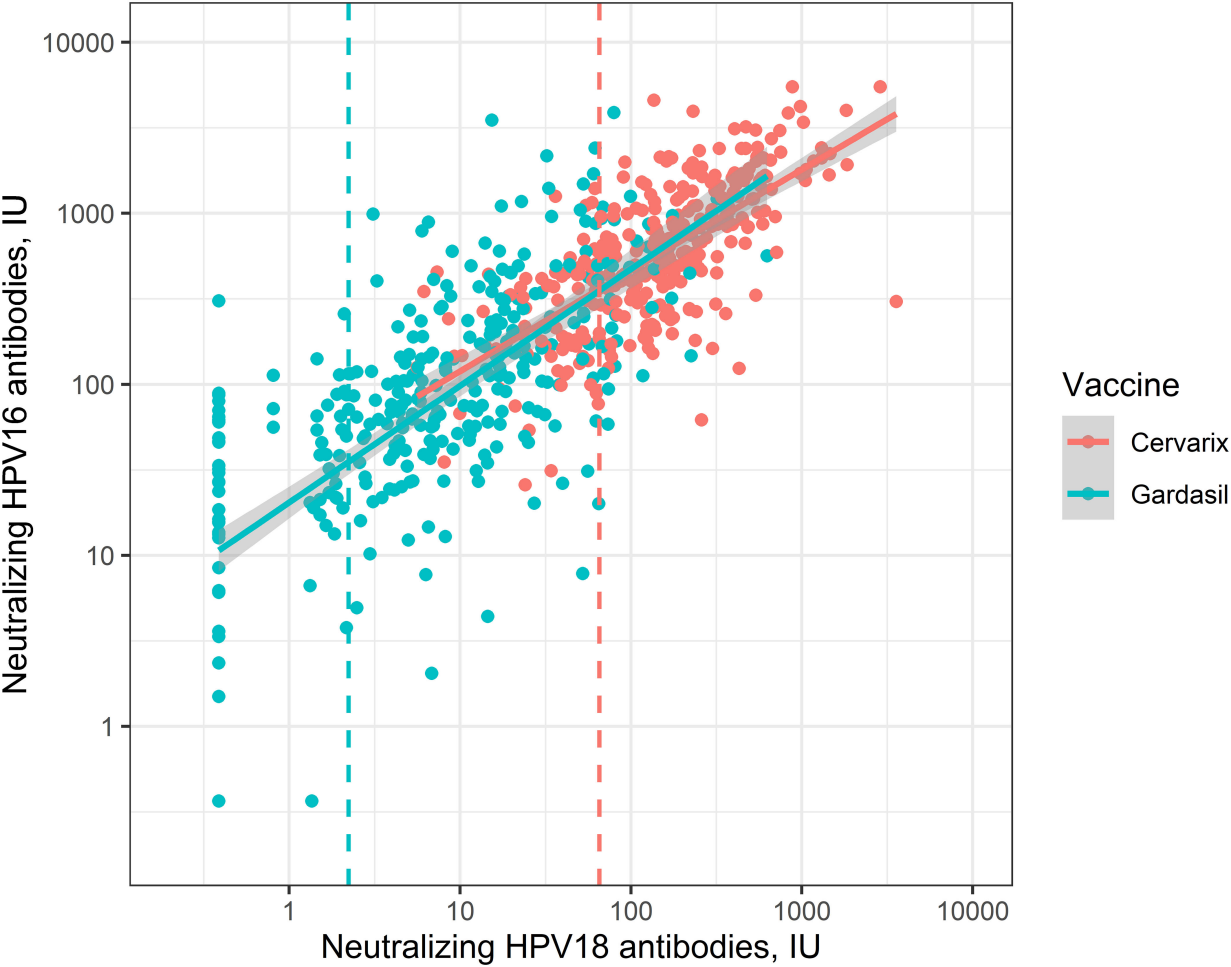
Neutralizing human papillomavirus (HPV) type 16 and type 18 antibody levels (International Units, IU/mL) in women vaccinated with three doses of the quadrivalent (HPV6/11/16/18) (●) or bivalent (HPV16/18) (●) vaccines at age 16 to 17 years. Upper margins of the lowest quartiles by vaccine are marked with dotted lines. Spearman (*r*s) correlation between HPV16 and HPV18 antibody levels in the lowest quartile by vaccine (rsquadrivalent = 0.3, rsbivalent = 0.3).

Finally, we evaluated the correlation (*r*s) of vaccine-induced neutralizing HPV16 and HPV18 antibody levels in the quadrivalent and bivalent vaccine recipients (**Figure 2**). In general, correlation among the bivalent vaccine recipients was good (*r*s_bi_ = 0.8), and distinguishable from that seen among the quadrivalent vaccine recipients *r*s_quadri_ = 0.6) due to excellent correlation among the higher quartiles of the bivalent vaccine-induced HPV16 and HPV18 antibody levels. However, among the lowest quartile, both the quadrivalent and bivalent vaccine recipients showed poor correlation (*r*s_quadri_ = 0.3, *r*s_bi_ = 0.3) of the neutralizing HPV16 and HPV18 antibody levels.

## Discussion

We demonstrate equal 68% and 65% vaccine efficacies against CIN3+, an immediate precursor of cervical cancer, in the ITT analyses of the cluster-randomized cohorts of quadrivalent and bivalent vaccine recipients and concomitant unvaccinated controls after 15 years of passive cancer registry-based follow-up.

Our results show the first independent head-to-head comparison of similarly aged cohorts enrolled to two consecutive phase III vaccine trials (FUTURE II and PATRICIA) in 2002–2003 and 2004–2005 on essentially overlapping 7 and 17 study sites around all the five Finnish county capitals with university hospitals. According to the Finnish legislation, the 16- to 17-year-old trial participants were able to consent independently without parental permission. Hence, owing to their young age, no sexual partner criteria were needed at enrollment to reduce the number of uninformative, baseline HPV positives (Lehtinen et al., 2006a, 2006b, 2006c). Sizeable adjacent birth cohorts of unvaccinated 18- to 19-year-olds, altogether 16,526 women, were enrolled all over the country in 2003 and 2005 (Lehtinen et al., 2006c). Various questionnaires have demonstrated that before age 23, virtually no opportunistic vaccination after the 2006 and 2007 licensure of the quadrivalent and bivalent vaccine took place in these women (Woodhall et al., 2011; Eriksson et al., 2013). Furthermore, crossed (post-trial) HPV vaccination after age 23 has had virtually no effect on CIN3+ rate (Rana et al., 2013).

The FCR gathers complete cancer information in Finland via the unique personal identifier (Pukkala et al., 2007) used also in this study, according to an informed consent also in this study. Thus, these individually and birth cohort (cluster) randomized cohorts of vaccinated and unvaccinated women formed an optimal basis for the long-term head-to-head comparison of VE against CIN3+. Earlier, this follow-up has shown sustainable 65% ITT VE against CIN3+ for the bivalent vaccine between 4 and 10 years post-vaccination (Lehtinen et al., 2012, 2017a).

Our independent head-to-head comparison has previously shown lack of HPV18 total pseudovirion and neutralizing post-vaccination antibody responses in up to 15% of the quadrivalent vaccine recipients but not in the bivalent vaccine recipients (Artemchuk et al., 2019; Mariz et al., 2021). We now demonstrate that among the lowest quartile of responders, both the quadrivalent and the bivalent vaccine recipients’ neutralizing HPV18 antibody levels lose material correlation with corresponding HPV16 antibody levels. This suggests that HPV vaccine-induced HPV18 antibody responses at the low end, i.e., in the low responders, are generally not optimal, and the definition of a protective vaccine-induced HPV18 antibody level is important. The altogether 20 cases with adenocarcinoma *in situ* and 3 cervical adenocarcinoma cases identified in the similarly aged unvaccinated controls indicated that these most notably HPV18-associated diseases are not infrequent among young adult women. This further emphasizes the need to define a protective HPV18 antibody level. Finally, however, it is important to note that eventually no difference between the ITT efficacies of the quadrivalent vs. bivalent vaccines against CIN3+ could be verified in this head-to-head comparison exploiting 15 years of passive cancer registry-based follow-up. The follow-up continues.

There are limitations in the enrollment and long-term follow-up of clinical vaccination trials. All 16- to 17-year-old female residents of the study site communities received an invitation letter with the official consent form. Information lectures were given on every secondary high school and technical school in the area (Lehtinen et al., 2006a, 2006b), yet it is possible that the most active and health-conscious adolescents participated the clinical trials. However, this may not have been the case for the unvaccinated participants of the control cohort. The consecutive conduction of the two clinical phase III trials 2 years apart enabled the combination of individual and birth cohort-wise (cluster) randomization, which, together with the country-wide FCR, helped to maintain the population-based nature of our study (Lehtinen et al., 2006c).

Our study is not a *post-hoc* analysis of two clinical trials but originally planned before the start of the phase III trials (Lehtinen et al., 2006a, 2006b, 2006c), albeit not initially powered for the evaluation of non-inferiority of the two vaccines in a head-to-head comparison. We have successfully used the same population-based design to show, for the first time, VE against invasive HPV-positive cancers in a randomized setting (Lehtinen et al., 2021) and a head-to-head comparison of the bivalent vs. quadrivalent or nonavalent vaccine immunogenicity (Artemchuk et al., 2019; Kann et al., 2021; Mariz et al., 2021; Arroyo et al., 2022). However, the population-based setting may diminish the international generalizability of the results.

In conclusion, we demonstrate the virtually identical ITT efficacy of the quadrivalent and bivalent vaccines, derived from the two lineages, against CIN3+. We will continue our passive follow-up for still another 5 to 10 years to ensure that no late-stage differences will emerge between the efficacy of the two vaccines.

## Data Availability

The original contributions presented in the study are included in the article/supplementary material. Further inquiries can be directed to the corresponding author.
